# A Review of Top‐Down Strategies for the Production of Quantum‐Sized Materials

**DOI:** 10.1002/smsc.202300086

**Published:** 2023-11-14

**Authors:** Zhexue Chen, Ce Zhao, Xuanping Zhou, Liuyang Xiao, Zhangqiang Li, Yong Zhang

**Affiliations:** ^1^ CAS Key Laboratory of Nanosystem and Hierarchical Fabrication CAS Center for Excellence in Nanoscience National Center for Nanoscience and Technology Beijing 100190 P. R. China; ^2^ University of Chinese Academy of Sciences Beijing 100049 P. R. China

**Keywords:** quantum dots, quantum sheets, quantum size effect, top-down

## Abstract

Nanoscience and technology have made significant achievements in the past few decades. Quantum‐sized materials, as a key component of nanomaterials, have attracted increasing interest due to their unique structures and extremely reduced sizes. Such fascinating materials have been widely applied in various fields because of their strong quantum confinement and remarkable surface/edge effects. Production methods have an important impact on the properties of quantum‐sized materials. Considering that many previous reviews have reported the synthesis of quantum‐sized materials by bottom‐up methods, this review will focus on the top‐down methods. The advantages and disadvantages of each strategy are analyzed. At the end, the perspectives and challenges toward the future development of quantum‐sized materials are discussed.

## Graphical abstract

Quantum‐sized materials have attracted much attention due to their surface/edge and quantum confinement effects. This review summarizes the latest advances in the production of quantum‐sized materials by top‐down strategies. The advantages and disadvantages of each method and the outlook of top‐down methods are discussed.

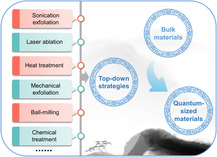

## 1 Introduction

The emergence of low‐dimensional nanomaterials has brought revolutionized development in advanced interdisciplinary science.^[^ ^1^, ^2^, ^3^ ^]^ Numerous types of novel low‐dimensional nanomaterials have been explored, such as two‐dimensional (2D) layered inorganic materials,^[^ ^4^, ^5^ ^]^ metal–organic fameworks (MOFs) and covalent organic frameworks (COFs),^[^ ^6^, ^7^ ^]^ perovskite nanomaterials,^[^ ^8^, ^9^ ^]^ and so on. It is well‐known that the term of nanoscale is crucial for low‐dimensional nanomaterials, as the nanoeffects caused by the size‐reduction will distinguish them from bulk materials. In particular, quantum‐sized materials with extremely exposed surfaces/edges have attracted much attention owing to their unique properties. Such fascinating materials have been widely applied in various fields, such as quantum information,^[^ ^10^ ^]^ light‐emitting diodes,^[^ ^11^ ^]^ solar cells,^[^ ^12^ ^]^ catalysis,^[^ ^13^ ^]^ energy storage,^[^ ^14^ ^]^ biomedical,^[^ ^15^ ^]^ and so on.

Both bottom‐up and top‐down methods have been employed for the production of quantum‐sized materials. The former usually starts from the bottom (i.e., atoms or molecules) to synthesize quantum‐sized materials under chemical interactions, while the latter involves tailoring the bulk materials into quantum‐sized materials by breaking their internal chemical bonds. Recently, many reviews have discussed the quantum dots derived from 2D materials, such as graphene,^[^ ^16^ ^]^ boron nitride (BN),^[^ ^17^ ^]^ black phosphorus (BP),^[^ ^18^ ^]^ transition metal dichalcogenide (TMD),^[^ ^19^ ^]^ and transition metal carbide and nitride (MXene).^[^ ^20^ ^]^ Most efforts have been devoted to the synthesis of quantum‐sized materials by bottom‐up methods.^[^ ^21^ ^]^ However, the production of quantum‐sized materials (not limited to 2D quantum dots) through top‐down methods has not been systematically summarized until now.

In this review, we focus on the latest research achievements in the production of quantum‐sized materials through top‐down strategies. First, we elaborated on the fundamental concepts of quantum‐sized materials and their effects. Then, top‐down approaches toward quantum‐sized materials were reviewed. Meanwhile, the advantages and limitations of each method were discussed. It should be emphasized that our research group has recently developed a general strategy toward the production of 2D quantum sheets (QSs) and 0D quantum dots (QDs) from bulk layered/nonlayered materials.^[^ ^22^, ^23^, ^24^, ^25^, ^26^, ^27^, ^28^, ^29^, ^30^, ^31^ ^]^ We introduced the unparalleled advantages of our method (i.e., the combination of silica‐assisted ball‐milling and sonication‐assisted solvent exfoliation), which would accelerate the establishment of a complete database/library of quantum‐sized materials. Finally, the current challenges and future perspectives of producing quantum‐sized materials by top‐down methods were presented.

## 2 Quantum‐Sized Materials

### 2.1 The Concepts of Quantum‐Sized Materials

Quantum‐sized materials have emerged as a fascinating class of nanomaterials with special and tunable properties, whose size is usually between 1 and 20 nm. The radius (rather than diameter or size) of a quantum‐sized material should be close to or less than its exciton Bohr radius.^[^ ^32^ ^]^ The formula for calculating the Bohr radius of excitons is as follows^[^ ^33^ ^]^

(1) $$R_{B}=\varepsilon (\frac{m_{0}}{\mu })a_{0}$$

**Figure 1:**
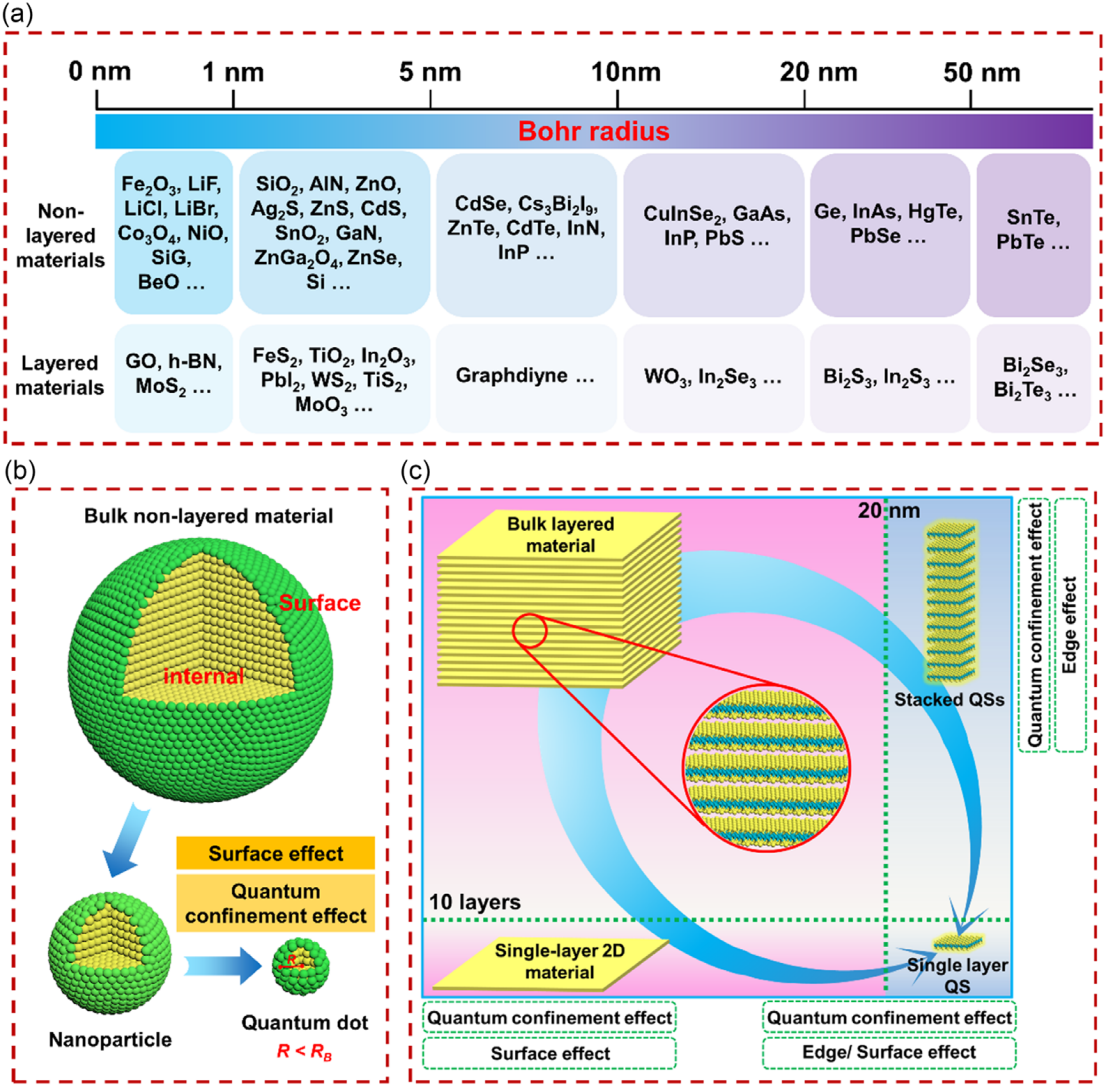
Quantum‐sized materials and their effects. a) Exciton Bohr radius. b) 0D QDs derived from bulk nonlayered materials. c) 2D QSs derived from bulk layered materials.

Where *R*_B_ is the exciton Bohr radius, *ε* is the dielectric constant of material, *m*_0_ is the free electron mass, *μ* ($\mu \;=\;\frac{m_{e}\cdot m_{h}}{m_{e}{+m}_{h}}$) is the reduced mass of the exciton, $m_{e}$ and $m_{h}$ are the masses of electrons and holes, respectively, $a_{0}$ is Bohr radius (0.53 Å). Notably, the dielectric constant of quantum‐sized materials is different from those in bulk. For example, the *ε* of tungsten disulfide (WS_2_) depends on the number of layers, which ranges from 5 to 14 when the thickness decreases.^[^ ^34^ ^]^ For WS_2_, $m_{e}$ = 0.33$m_{0}$, $m_{h}$ = 0.43$m_{0}$, *μ *≈ 0.2$m_{0}$,^[^ ^35^ ^]^ when *ε* = 14, the *R*_B_ is 3.7 nm. We have summarized the exciton Bohr radii of typical layered and nonlayered materials, as shown in **Figure** **1**a. Obviously, the *R*_B_ of different materials is quite different and spans a wide range (from a fraction of a nanometer to several tens of nanometers). For most materials, the *R*_B_ is less than 10 nm, so the size between 1 and 20 nm is generally considered as quantum scale.

According to dimensionality, quantum‐sized materials could be classified into 0D QDs, one‐dimensional quantum rods (1D QRs) and 2D QSs. 0D QDs means that the size of the materials in all three dimensions is not larger than twice of their exciton Bohr radius. Typical examples include metal quantum dots (e.g., AuQDs,^[^ ^36^ ^]^ AgQDs),^[^ ^37^ ^]^ semiconductor quantum dots (e.g., SiQDs,^[^ ^38^ ^]^ Ag_2_S QDs),^[^ ^39^ ^]^ alloy quantum dots (e.g., FeNi QDs,^[^ ^40^ ^]^ NiCoFePtRh QDs),^[^ ^41^ ^]^ and so on. The shape of quantum dots is generally relatively regular, known as spherical, cubic, or triangular. Although the sizes of 1D QRs and 2D QSs are also smaller than their exciton Bohr diameter in all three dimensions, they differ from 0D QDs in that the former has rod‐shaped properties and the latter possesses layered properties. The anisotropy in their geometric structure would result in different properties or effects.^[^ ^42^ ^]^ For the synthesis of 1D QRs, most of the studies focus on the growth of rod‐shaped semiconductors using a surfactant‐controlled growth mode, such as CdSe QRs,^[^ ^43^ ^]^ ZnO QRs,^[^ ^44^ ^]^ and so on. The term “quantum sheets” first appeared in a report by German scientists.^[^ ^45^ ^]^ However, researchers usually pay little attention to distinguishing QSs from QDs, and habitually call QSs as QDs.^[^ ^46^, ^47^, ^48^ ^]^ Actually, 2D QSs are produced from layered materials while 0D QDs are produced from nonlayered materials. It should be noted that 2D QSs are derived from the continuous development and intersection of two‐dimensional materials and quantum systems. Generally, the lateral size of a QS is less than 20 nm and the thickness is below 10 layers. Typical 2D QSs include graphene QSs (GQSs),^[^ ^23^ ^]^ boron nitride QSs (BNQSs),^[^ ^23^ ^]^ black phosphorus QSs (BPQSs),^[^ ^49^ ^]^ molybdenum disulfide QSs (MoS_2_ QSs),^[^ ^22^ ^]^ and so on.

### 2.2 The Effects of Quantum‐Sized Materials

Various new characteristics in optics,^[^ ^50^ ^]^ electronics,^[^ ^51^ ^]^ magnetism,^[^ ^52^ ^]^ and thermology^[^ ^53^ ^]^ were generated in quantum‐sized materials thanks to the strong surface/edge effects, quantum confinement effects, and macroscopic quantum tunneling effects.^[^ ^54^, ^55^, ^56^, ^57^, ^58^ ^]^ Specifically, the surface/edge effects mean changes in the physical and chemical properties of materials with their size‐reduction. The periodic boundary conditions of crystals will be destroyed when the size of nanoparticles is equal to or less than a certain physical characteristic size, such as the wavelength of light, the average free path of electrons, the exciton Bohr diameter, and the transmission depth, resulting in unique phenomena. Furthermore, the proportion of atoms (lattices) on the surface/edge of the quantum‐sized materials will sharply increase and dominate its performance as the size decreases to the quantum scale.^[^ ^59^, ^60^ ^]^ Quantum confinement effects: When the size of a semiconductor material or metal is down to the quantum scale, the energy levels of the material will change from continuous to discrete, and the energy band will be broadened.^[^ ^56^, ^57^ ^]^ For example, Ozin et al. synthesized eight 0D SiQDs with controlled sizes (1–2 nm) through chemical synthesis and presented size‐dependent photoluminescence colors (from green to red).^[^ ^61^ ^]^ Macroscopic quantum tunneling effects: When the total energy of a microparticle is less than the height of the potential barrier, the particle could still cross this potential barrier.^[^ ^62^, ^63^ ^]^ The macroscopic quantum tunneling effect is one of the most interesting phenomena in modern quantum physics.^[^ ^64^ ^]^

As shown in Figure 1b,c, the nanoeffect of the material will occur when the size decreases (from the bulk to the nanoscale and then to the quantum scale). For 0D QDs, the surface effects and quantum confinement effects are both prominent. For 2D QSs, it not only retains the intrinsic characteristic of two‐dimensional materials but also demonstrates strong (anisotropic) in‐plane and out‐of‐plane quantum confinement effects, as well as prominent edge effects.

## 3 Top‐Down Production of Quantum‐Sized Materials

**Figure 2:**
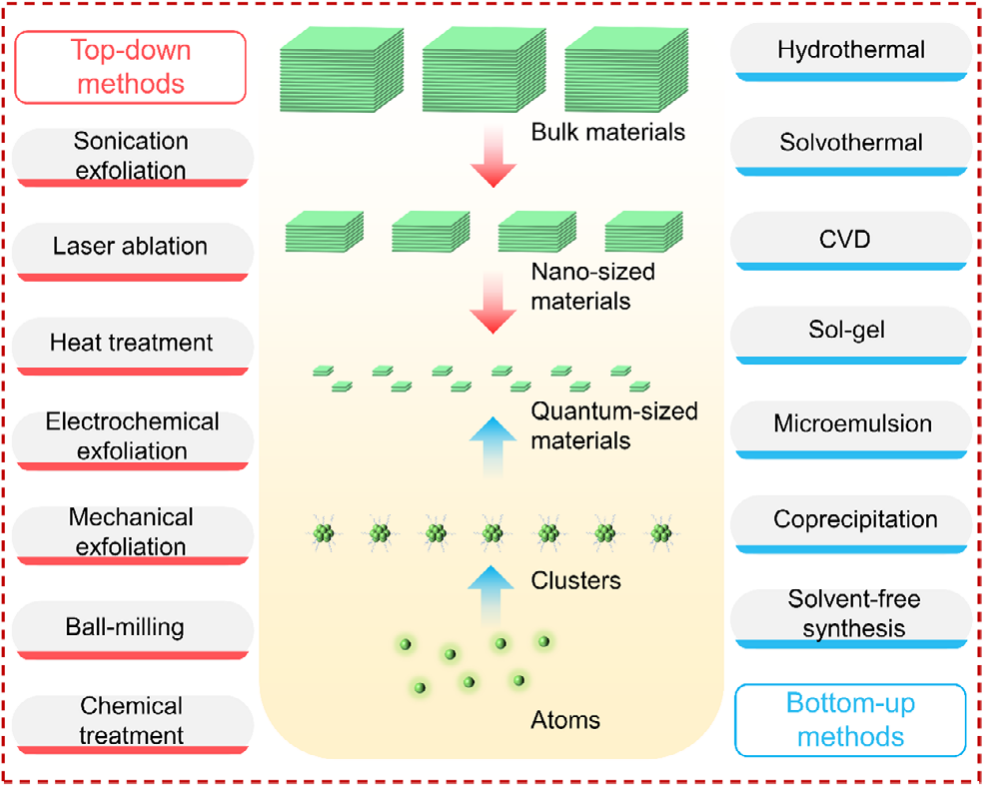
Top‐down and bottom‐up strategies for the production of quantum‐sized materials.

The production strategies of quantum‐sized materials could be divided into two categories: Bottom up and top down, as shown in **Figure** **2**. The bottom‐up methods involve the assembly of the bottom (i.e., atoms or molecules) to synthesize the required materials through various precursors under chemical interaction, such as hydrothermal/solvothermal,^[^ ^65^, ^66^ ^]^ chemical vapor deposition (CVD),^[^ ^67^ ^]^ sol–gel,^[^ ^68^ ^]^ microemulsion,^[^ ^69^ ^]^ coprecipitation,^[^ ^70^ ^]^ and so on.^[^ ^71^ ^]^ These methods have been widely applied to prepare nanomaterials with the advantage of accurately controlling the size, morphology, and surface functionalization of the material. However, current bottom‐up methods suffer from either rigorous conditions or tedious posttreatment. In addition, the surface of nanomaterials synthesized by bottom‐up methods was thermodynamically stable (equilibrium state) due to the process driven by Gibbs free energy.^[^ ^72^ ^]^

Considering the detailed bottom‐up methods in previous reviews,^[^ ^73^, ^74^, ^75^ ^]^ herein, we will only focus on the top‐down methods for the production of quantum‐sized materials. The top‐down methods usually tailor bulk materials into quantum‐sized materials through physical strategies, such as sonication exfoliation,^[^ ^76^ ^]^ laser ablation,^[^ ^77^ ^]^ heat treatment,^[^ ^78^ ^]^ mechanical exfoliation,^[^ ^79^ ^]^ and so on.^[^ ^80^ ^]^ **Table** **1** summarizes typical top‐down methods for the preparation of quantum‐sized materials.

**Table 1:** Summary of top‐down strategies toward quantum‐sized materials

Material	Method	Lateral size [nm]	Thickness [nm]	Yield [wt%]	References
Graphene QSs	One‐step ultrasonication	3	0.7–3	/	[196]
	electrochemical oxidation in ammonia	4.7	3–8	28	[197]
	ionic liquid‐assisted electrochemical exfoliation	2.4	0.7–2.8	/	[143]
	cryo‐mediated pretreatment and liquid‐phase exfoliation	1.71	0.6	1	[130]
	silica‐assisted ball‐milling and ultrasonication	2.9	0.5	44.6	[24]
	microwave‐assisted	2–7	0.5–2	8	[141]
	silica‐assisted ball‐milling and ultrasonication	3.2	0.5	35.5	[23]
Graphene NSs	Scotch‐tape method	1000–8000	0.5–2.0	≈100	[167]
Carbon QDs	Dual‐beam pulsed laser ablation	3.8	2	/	[113]
h‐BN QSs	Mechanochemical exfoliation in ethanol	2–6	1–2	/	[170]
	femtosecond laser ablation	0.5–2.5	/	/	[198]
	solvothermal treatment	1.91–3.23	2.41	/	[199]
	silica‐assisted ball‐milling and ultrasonication	1.9	0.6	33.6	[23]
BP QSs	Probe sonication with bath sonication in NMP	2.6	1.5	/	[49]
	Solvothermal in NMP	2.1	/	/	[135]
	Mechanical blending	2.25	0.58–1.45	/	[165]
	electrochemical exfoliation and synchronous fluorination	5.0	2.0	/	[159]
MoS_2_ QSs	Chemical intercalation and ultrasonication	3.5	0.5–1.2	11	[200]
	ultrasonication and solvothermal	3.3	1.2	13	[90]
	Solvothermal in ethanol	2.9	1.4–2.8	/	[201]
	electrochemically induced etching	5	0.7–1.4	/	[158]
	reflux pretreatment and bath sonication treatment	2–6	0.6–1	1.5	[78]
	ultrathin cutting and ultrasonication	3–6	0.7–1.8	40	[166]
	salt‐assisted ball‐milling and ultrasonication	4.9	1.0	25.5	[22]
	silica‐assisted ball‐milling and ultrasonication	2.1	0.9	30.2	[23]
WS_2_ QSs	Cryo‐mediated pretreatment and liquid‐phase exfoliation	3.73	1.0	1	[130]
	ultrasonication and hydrothermal	2.5	0.8–1.0	5.3	[202]
	Pulsed‐laser ablation	5.8	0.6–1.3	/	[112]
	ultrasonication and solvothermal treatment	2.5	1.2	18	[90]
	salt‐assisted ball‐milling and ultrasonication	4.2	/	20.1	[22]
	silica‐assisted ball‐milling and ultrasonication	/	/	28.2	[23]
MoSe_2_ QSs	Grinding and sonication	2.7	1.8	/	[164]
g‐C_3_N_4_ QSs	Acid treatment and hydrothermal treatment	4	0.35	/	[186]
S QDs	H_2_O_2_‐assisted etching	5.0/3.5	/	/	[187]
MXene/MAX QSs	Acoustomicrofluidic	10.7	1.1	7	[97]
	silica‐assisted ball‐milling and ultrasonication	2.8	1.3	15.6	[29]
	silica‐assisted ball‐milling and ultrasonication	2.6	1.1	17.8	[29]
	Hydrothermal with heteroatom functionalization	2.73	0.85	/	[136]
ZnSe QDs	Femtosecond pulsed‐laser ablation	4–6	/	/	[115]
PbS QDs	Silica‐assisted ball‐milling and ultrasonication	6.9	6.5	>15	[27]
CdS QDs	Silica‐assisted ball‐milling and ultrasonication	4.9	4.7,	>15	[27]
CuS QDs	Silica‐assisted ball‐milling and ultrasonication	5.1	4.9	>15	[27]
FeS QDs	Silica‐assisted ball‐milling and ultrasonication	3.3	3.1	35.6	[27]
ZnS QDs	Silica‐assisted ball‐milling and ultrasonication	3.3	3.3	>15	[27]

### 3.1 Sonication Exfoliation

**Figure 3:**
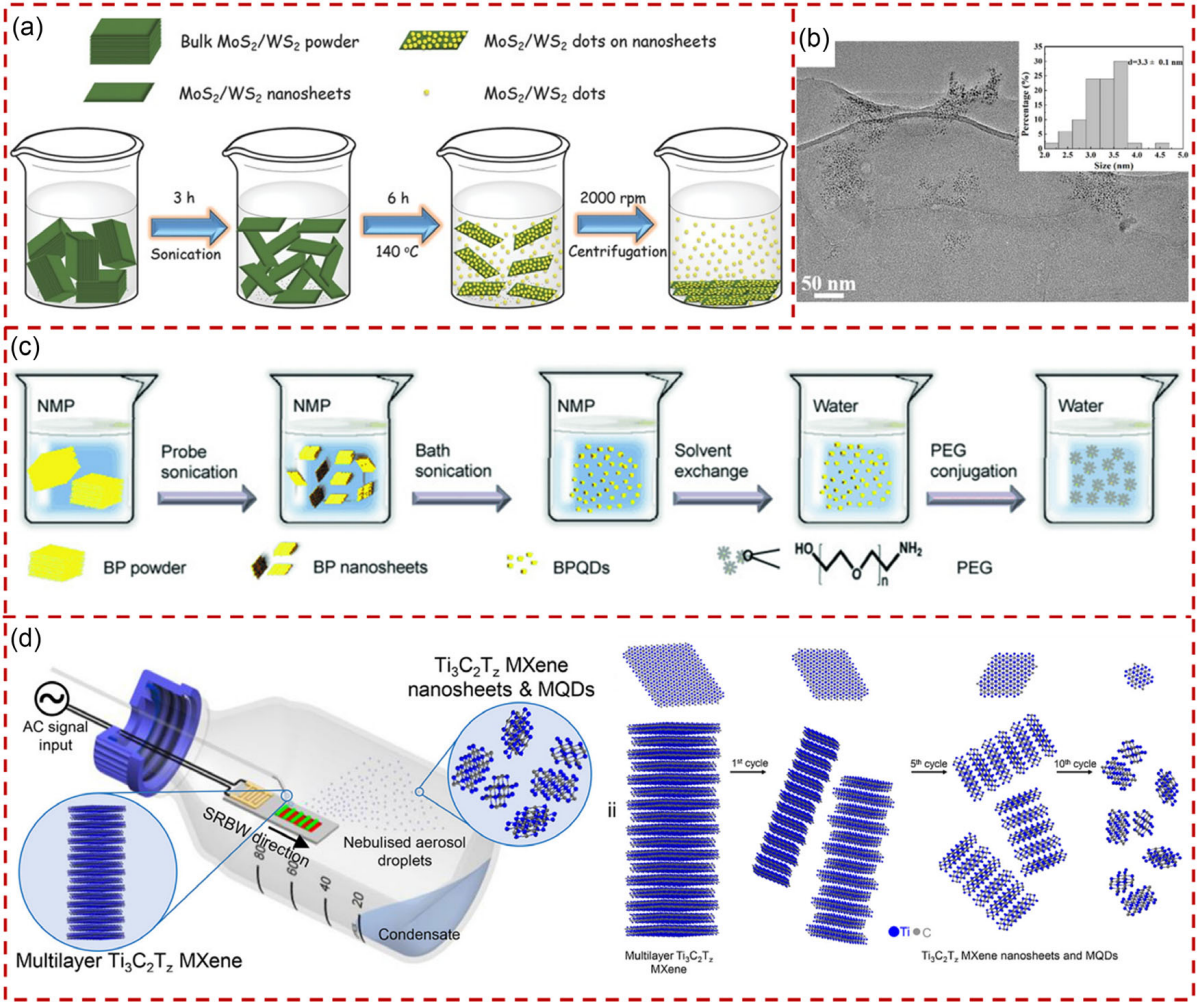
The production of quantum‐sized materials through sonication exfoliation. a) Schematic illustration of the production process of MoS_2_/WS_2_ QSs by using a liquid phase exfoliation and solvothermal treatment. b) Corresponding TEM images of MoS_2_ QSs prepared with the treatment of (a). a,b) Reproduced with permission.^[^ ^90^ ^]^ Copyright 2015, Wiley‐VCH. c) Schematic illustration of the preparation and surface modification of BPQSs through probe sonication and bath sonication. Reproduced with permission.^[^ ^49^ ^]^ Copyright 2015, Wiley‐VCH. d) Schematic illustration of the acoustomicrofluidic fabrication of Ti_3_C_2_T_*z*_ MXene NSs/QSs. Reproduced with permission.^[^ ^97^ ^]^ Copyright 2021, American Chemical Society.

As early as 2008, Coleman et al. produced 2D nanosheets (NSs) from layered bulk materials (e.g., graphene, TMDs) by using sonication‐assisted liquid phase exfoliation (LPE).^[^ ^81^, ^82^ ^]^ Nowadays, sonication‐assisted LPE has become a commonly used method for producing 2D QSs from bulk layered materials.^[^ ^76^, ^83^, ^84^, ^85^, ^86^ ^]^ This process generally utilizes the hydrodynamic forces generated by ultrasound in an appropriate solvent to realize exfoliation. To obtain more uniform quantum‐sized materials, other methods such as microwave and thermal treatment need to be combined.^[^ ^87^, ^88^, ^89^ ^]^ For instance, Wu et al. produced MoS_2_ and WS_2_ QSs through the combination of sonication and thermal treatment.^[^ ^90^ ^]^ The schematic illustration of the production process of MoS_2_/WS_2_ QSs is shown in **Figure** **3**a. The nanosheets were obtained by sonication of the bulk material in *N,N*‐dimethylformamide (DMF) for 3 h, followed by sonication of the corresponding NSs under heating conditions (140 °C) for 6 h, and finally centrifuged to obtain MoS_2_/WS_2_ QSs. The transmission electron microscopy (TEM) observation with size distribution of MoS_2_ QSs is presented in Figure 3b. The resulting MoS_2_ and WS_2_ QSs possess monolayer thickness with an average size about 3.3 and 2.5 nm, respectively. This strategy could become a general and green method for producing 2D TMD QSs.

In the process of sonication‐assisted LPE, it is essential to choose suitable solvents to match the surface energy. To ensure that the quantum‐sized materials could be well dispersed in solvents, 1‐methyl‐2‐pyrrolidone (NMP), DMF, or mixed solvents were used frequently.^[^ ^91^ ^]^ In addition, the polarity of the solvent needs to be considered for different materials.^[^ ^92^ ^]^ Chu et al. prepared BPQSs from bulk BP powder in NMP with a LPE approach, which included probe sonication with bath sonication, as shown in Figure 3c.^[^ ^49^ ^]^ After sonication and centrifugation, the as‐obtained BPQSs were distributed in water, and the PEG conjugation was introduced to improve the stability in the physiological medium. Compared with each method alone, the combination of probe sonication and bath sonication allowed for more effective preparation of high‐quality BPQSs with an excellent near‐infrared (NIR) photothermal performance, which is promising for application in targeted photothermal cancer therapy. Ajayan et al. used acetonitrile/isopropanol (IPA) as solvents for LPE to break bulk TiS_2_ powder into TiS_2_ QS.^[^ ^93^ ^]^ Compared with bulk TiS_2_ and TiS_2_ NSs, the as‐produced TiS_2_ QSs exhibited higher hydrogen evolution reaction (HER) activity owing to their extremely exposed edge sites.

Recently, a class of nanometer‐amplitude high‐frequency (MHz order) hybrid sound waves, named as surface‐reflected bulk waves (SRBW), has been discovered and proven to effectively exfoliate bulk layered materials.^[^ ^94^, ^95^, ^96^ ^]^ Yeo et al. reported a chemical‐free acoustomicrofluidic preparation of high‐purity Ti_3_C_2_T_z_ MXene QSs in an SRBW device at room temperature.^[^ ^97^ ^]^ The schematic diagram of the experimental setup and the preparation process of MXene NSs and QSs are shown in Figure 3d. The multilayer Ti_3_C_2_T_z_ MXene was delaminated into monolayer structure under the large mechanical force generated by high‐frequency acoustic nebulization. Then, MXene QSs were produced with lower oxygen content, excellent photoluminescence, and electrochemical performances after multiple nebulization cycles. Unlike hydrothermal/solvothermal^[^ ^98^, ^99^, ^100^ ^]^ or electrochemical^[^ ^101^ ^]^ methods, the acoustomicrofluidic method was chemical‐free with mild conditions and fast processes.

Based on sonication, researchers have successfully produced 2D QSs with relatively high quality.^[^ ^93^, ^98^ ^]^ However, sonication exfoliation still has shortcomings, such as low yield, poor controllability, surface oxidation, and so on. This was mainly attributed to the extremely high local temperatures, ultra‐high pressures, and sudden cooling/heating changes caused by the ultrasonic cavitation process. There are several ways to reduce or avoid these issues, such as adjusting the ultrasonic frequency and intensity, changing the chemical composition and temperature of the working fluid, and optimizing the ultrasonic control parameters.^[^ ^102^ ^]^

### 3.2 Laser Ablation

The pulsed laser ablation (PLA) technology, as a facile and eco‐friendly physical method, has shown great potential in “green” fabrication of nanomaterials.^[^ ^103^, ^104^, ^105^, ^106^, ^107^, ^108^ ^]^ In 2001, Aya et al. obtained high‐purity silicon QDs with a size of less than 10 nm by PLA in a low‐pressure inert gas.^[^ ^109^ ^]^ Khakani et al. reported a direct PLA approach for producing PbS QDs in a helium atmosphere without chemical or posttreatment.^[^ ^110^ ^]^ Through PLA, PbS QDs with controlled size and surface density could be directly laser‐deposited on single‐wall carbon nanotubes (SWCNTs), thereby, achieving efficient and rapid charge transfer between the PbS QDs and SWCNTs. However, the development and application of PLA technology were limited due to the expensive equipment, strict conditions, poor operability, and practicality.

**Figure 4:**
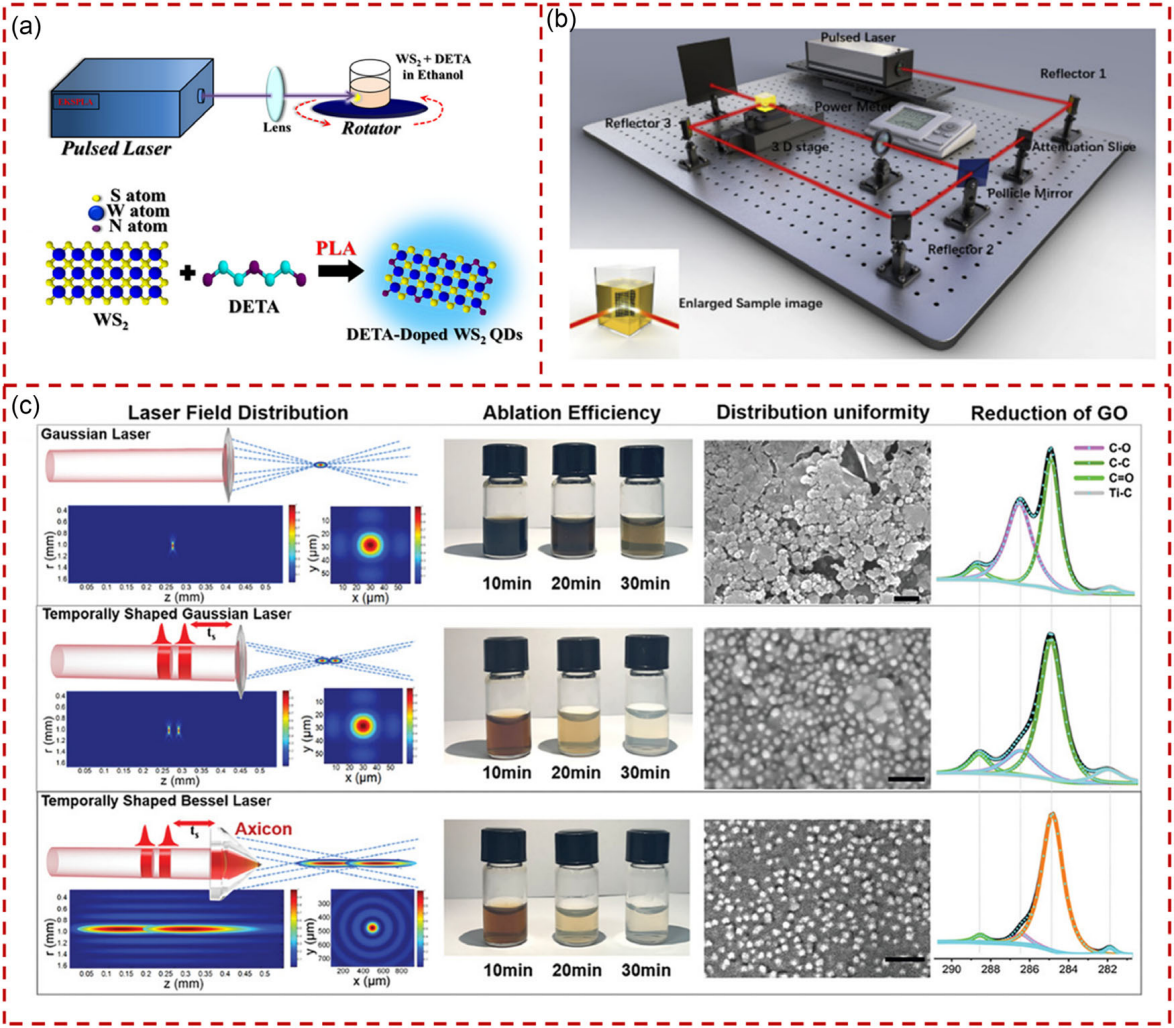
The production of quantum‐sized materials through laser ablation. a) Schematic illustration of the experimental setup for pulsed laser ablation and the preparation process of the WS_2_ QSs doped with diethylenetriamine. Reproduced with permission.^[^ ^112^ ^]^ Copyright 2018, American Chemical Society. b) Schematic illustration of the production process of carbon quantum dots by dual‐beam pulsed laser ablation. Reproduced with permission.^[^ ^113^ ^]^ Copyright 2020, Elsevier. c) Schematic diagram of three different types of laser processing MXene QSs in GO dispersion solution, the contrast photographs, SEM images (scale bar: 20 nm), and X‐ray photoelectron spectroscopy characterization were displayed. Reproduced with permission.^[^ ^114^ ^]^ Copyright 2022, Wiley‐VCH.

Recently, pulsed laser ablation in liquid (PLAL) has attracted more attention owing to convenient processing and mild conditions.^[^ ^111^ ^]^ Shen et al.^[^ ^112^ ^]^ demonstrated that controllable doping of WS_2_ QSs was achieved via PLAL, where diethylenetriamine (DETA) as the dopant. **Figure** **4**a illustrates the experimental setup for producing WS_2_ QSs doped with DETA. The current modulation, carrier concentration, and field‐effect mobility of doped WS_2_ QSs (about 6 nm) were greatly enhanced with the introduction of DETA during PLA. Sun et al. produced homogeneous carbon QDs (CQDs) by ultrafast and highly efficient dual‐beam PLA from low‐cost carbon cloth, as shown in Figure 4b.^[^ ^113^ ^]^ A single laser beam was divided into two laser beams for shortening the laser ablation time, and the as‐produced CQDs presented high photoluminescence quantum yield (PLQY) (i.e., 35.4%). Qu et al. reported an in situ strategy employing a temporally and spatially shaped (Bessel) laser (TSBL) for synthesizing MXene QSs.^[^ ^114^ ^]^ A transparent composite electrode of uniformly distributed MXene QSs attached to few‐layered graphene oxide (GO) could be successfully prepared by ablating a MXene target immersed in a GO dispersion through TSBL. Figure 4c shows the simulation of light fields in three laser focusing modes, as well as the control photographs of the MXene QSs/GO solution, the scanning electron microscopy images, and X‐Ray photoelectron spectroscopy characterization. This study indicates that TSBL not only has a wider light field distribution but also could greatly improve the manufacturing efficiency of MXene QSs/GO.

The development of PLA has roughly undergone five stages according to the pulse width of the laser:^[^ ^108^, ^115^, ^116^ ^]^ millisecond laser ablation,^[^ ^117^, ^118^ ^]^ microsecond laser ablation,^[^ ^119^ ^]^ nanosecond laser ablation,^[^ ^120^, ^121^, ^122^ ^]^ picosecond laser ablation,^[^ ^123^, ^124^ ^]^ and femtosecond laser ablation.^[^ ^115^, ^125^ ^]^ Unlike ultrafast lasers, millisecond or microsecond pulsed lasers have a relatively low power density and typically cause linear absorption and joule heating of the target. Therefore, the quantum‐sized materials with larger sizes and narrower bandgaps than photon energy could be heated or even evaporated, while those with smaller sizes and wider bandgaps remain intact, allowing for the preparation of quantum‐sized materials with narrow size distributions.^[^ ^126^ ^]^ Actually, it is possible to control the size and performance of quantum‐sized materials by adjusting laser parameters (pulse width, pulse repetition rate, working wavelength, pulse energy, etc.), ablation time, and solvent type during the PLA experiment.^[^ ^127^, ^128^, ^129^ ^]^ Although the laser ablation method was facile and environmentally friendly compared with chemical treatment, its expensive cost and poor repeatability need to be improved in the future.^[^ ^106^ ^]^

### 3.3 Heat Treatment

**Figure 5:**
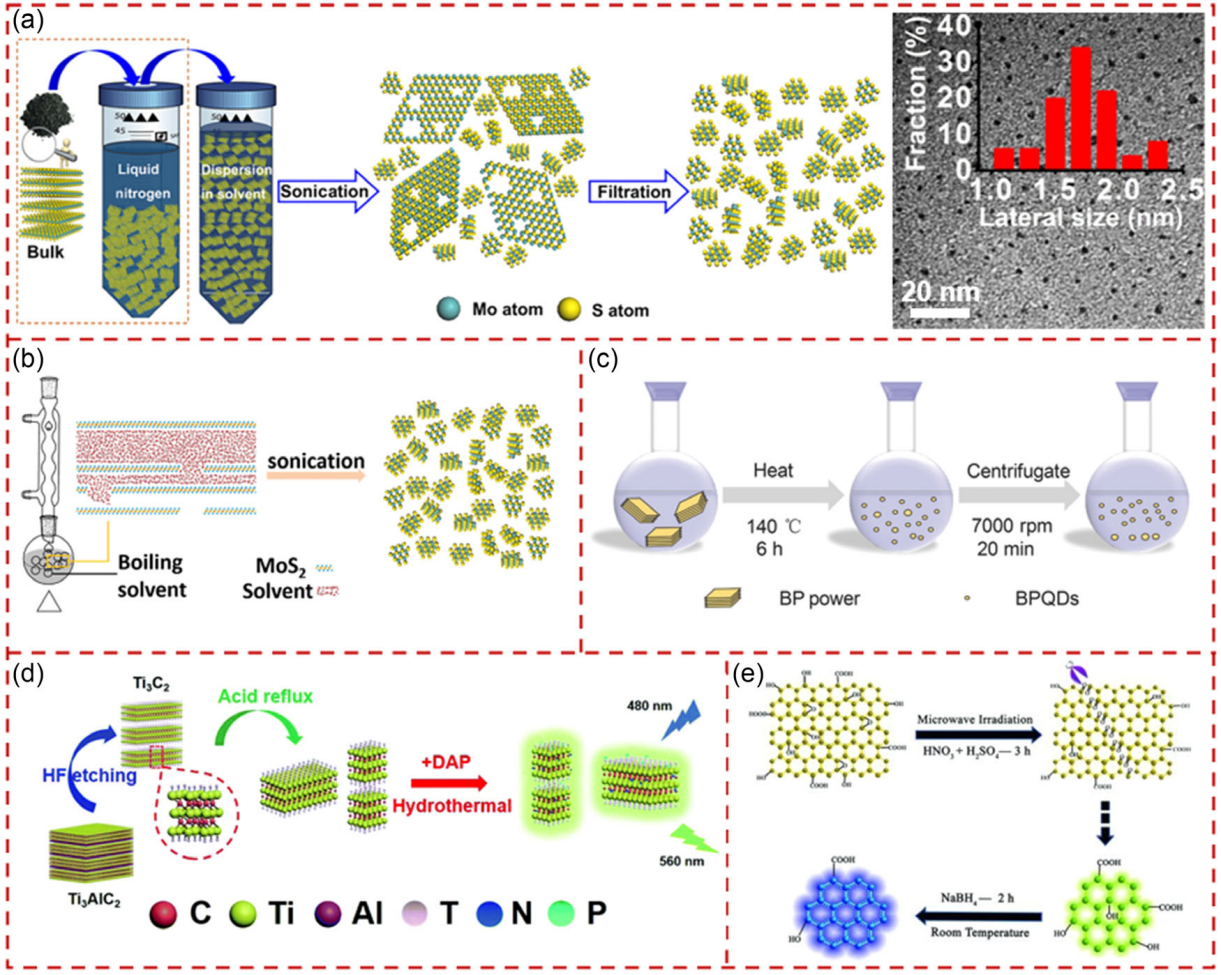
The production of quantum‐sized materials through heat treatment. a) Schematic illustration of the cryo‐exfoliation process for the production of MoS_2_ QSs and corresponding TEM images. Reproduced with permission.^[^ ^130^ ^]^ Copyright 2017, The Authors, published by American Association for the Advancement of Science. From ref. [130]. © The Authors, some rights reserved; exclusive licensee AAAS. Distributed under a CC BY‐NC 4.0 license http://creativecommons.org/licenses/by-nc/4.0/. Reprinted with permission from AAAS. b) Schematic illustration of the reflux pretreatment and bath sonication to fabricate MoS_2_ QSs. Reproduced with permission.^[^ ^78^ ^]^ Copyright 2019, Elsevier. c) Schematic illustration of the solvothermal approach for the production of BPQSs. Reproduced with permission.^[^ ^135^ ^]^ Copyright 2016, Wiley‐VCH. d) Schematic illustration of the hydrothermal approach for the production of N, P‐MXene QSs. Reproduced with permission.^[^ ^136^ ^]^ Copyright 2019, Royal Society of Chemistry. e) Schematic illustration for the production of GQSs via a microwave‐assisted process. Reproduced with permission.^[^ ^141^ ^]^ Copyright 2012, Wiley‐VCH.

Heat treatment has been widely used to produce quantum‐sized materials, including low‐temperature treatment and high‐temperature treatment. For low‐temperature treatment, small cracks could be generated in bulk materials through cryo‐mediated method, followed by sonication treatment to fabricate quantum‐sized materials. Ajayan et al. exhibited a facile method based on cryo‐mediated pretreatment (i.e., liquid nitrogen pretreatment) and LPE for producing MoS_2_ and WS_2_ QSs in a short time, as presented in **Figure** **5**a.^[^ ^130^ ^]^ Atomically thin MoS_2_ QSs with lateral sizes ranging from 0.78 to 2.4 nm and WS_2_ QSs with lateral sizes ranging from 2.18 to 5.99 nm were prepared via pretreatment of the layered material powders in liquid nitrogen then sonication exfoliation in IPA/H_2_O solution. Moreover, the developed method could employ a wide range of common liquids as solvents and was suitable for producing 2D QSs from various layered materials. Their group also developed a high‐temperature pretreatment method (i.e., reflux pretreatment) to prepare 2D QSs.^[^ ^78^ ^]^ As shown in Figure 5b, the bulk MoS_2_ powders were dispersed in IPA/H_2_O (7:3 in volume) and refluxed for 24 h at 84 °C. During the reflux pretreatment, the boiling solvent was inserted MoS_2_ layers to disrupt the interlayer interaction forces. After that, the as‐treated MoS_2_ powders were subjected to sonication and cascade centrifugation to obtain MoS_2_ QSs (with lateral size of 2–6 nm).

Hydrothermal/solvothermal was widely applied for preparing QSs, either chemically or physically.^[^ ^131^, ^132^, ^133^, ^134^ ^]^ In terms of physical production, Yu et al. produced BPQSs from the bulk BP crystals via the solvothermal approach in NMP, as shown in Figure 5c.^[^ ^135^ ^]^ The bulk BP powders were dispersed into saturated NaOH/NMP solution with vigorous and continuous stirring for 6 h at 140 °C under nitrogen atmosphere. BPQSs with an average size of 2.1 nm were finally produced following the centrifugation and separation of the resulting suspensions for 20 min at 7000 rpm. Xu et al. successfully prepared the nitrogen and phosphorous functionalized Ti_3_C_2_ MXene QSs (N, P‐MXene QSs) from the bulk Ti_3_C_2_ through hydrothermal method, as presented in Figure 5d.^[^ ^136^ ^]^ First, Ti_3_AlC_2_ was used as the raw material to prepare Ti_3_C_2_ MXene NSs via a two‐step process of hydrofluoric acid (HF) etching and strong acid reflux. The Al layer was completely removed from Ti_3_AlC_2_ by HF etching, leading to the formation of layered Ti_3_C_2_ NS stacks. Such a layered structure of Ti_3_C_2_ makes it easy to be exfoliated, similar to the production of graphene by graphite exfoliation. Then, the obtained MXene NSs were subjected to hydrothermal treatment in diammonium phosphate (DAP) at 120 °C for 12 h to produce N, P‐MXene QSs. The N, P‐MXene QSs with a lateral size of around 2.73 nm showed green photoluminescence at 560 nm, and the PLQY reached 20.1%. Hydrothermal/solvothermal treatment could be regarded as an alternative strategy for preparing QSs, but it was only applicable to the layered materials with weak breaking strength. In addition, the intrinsic structure of the material will be severely damaged through hydrothermal/solvothermal treatment.

Microwave processing could be used as a heat treatment method due to the intense interaction between materials and microwave radiation, resulting in rapid local heating. In recent years, Microwave‐assisted fabrication has been utilized to produce organic and inorganic quantum‐sized materials thanks to its simplicity and efficiency.^[^ ^137^, ^138^, ^139^, ^140^ ^]^ Zhu et al. exhibited an efficient and eco‐friendly microwave‐assisted approach to fabricate stabilizer‐free greenish‐yellow GQSs (gGQSs) and brightly blue GQSs (bGQSs).^[^ ^141^ ^]^ The process of preparing gGQSs and bGQSs is shown in Figure 5e. The gGQSs were fabricated from graphene oxide (GO) nanosheets under microwave irradiation and acid conditions (HNO_3_ and H_2_SO_4_) within 3 h. The bGQSs were prepared by moderately reducing the gGQSs with NaBH_4_ within 2 h. The literal sizes of gGQSs were in the range of 2–7 nm and their thicknesses were ranging from 0.5 to 2 nm. Considering that the size of bGQS was basically the same as that of gGQS, the PL blueshift of bGQS could be attributed to surface/edge effects. Microwave heating was a noncontact heat transfer method that could complete the preparation of required materials in a short time compared to traditional heating strategies.^[^ ^142^ ^]^ Therefore, microwave heating could serve as a potential auxiliary method for preparing quantum‐sized materials.

### 3.4 Electrochemical Exfoliation

Electrochemical exfoliation of bulk materials (e.g., graphite,^[^ ^143^, ^144^, ^145^ ^]^ carbon fiber,^[^ ^143^ ^]^ graphene,^[^ ^146^ ^]^ MoS_2_,^[^ ^147^ ^]^ and BP)^[^ ^148^ ^]^ was an effective strategy for achieving high‐quality and uniform‐size QSs. In this approach, QSs were prepared by using bulk materials as anode and platinum electrode as the cathode in different acidic, alkaline, or salt solution electrolyte systems. Compared with chemical etching methods, electrochemical exfoliation showed great advantages because it rarely uses harsh chemical etchants. Besides, electrochemical exfoliation could selectively etch precursors by changing the applied electrical potential.

The electrochemical exfoliation of QSs was generally impacted by electrode precursors, types of electrolytes, water contents, and current density.^[^ ^149^, ^150^, ^151^ ^]^ Taking the production of GQSs as an example, a variety of electrode precursors could be used for the electrochemical exfoliation, such as graphite, graphene oxide, carbon fibers, and multiwalled carbon nanotubes (MWCNTs).^[^ ^152^, ^153^ ^]^ In 2011, Qu et al. reported an alternative electrochemical approach for direct preparation of functional GQSs with a size of 3–5 nm.^[^ ^154^ ^]^ The electrochemical exfoliation of GQSs was prepared in a 0.1 m phosphate buffer solution (PBS) with a filtration‐formed film of graphene as the working electrode. Pillai et al. synthesized 3, 5, and 8 nm GQSs at 1 V using a thin film of MWCNTs as the precursor, while propylene carbonate and LiClO_4_ as electrolytes.^[^ ^155^ ^]^ Owing to the high cost of graphene and MWCNTs precursor materials, cheap precursors (such as graphite rods, carbon fibers, and coke) have attracted much interest. For example, Wang et al. reported an economical production of multicolored fluorescence GQSs from a small cuboid coke with a mixture of (NH_4_)_2_S_2_O_8_, methanol, and water as the supporting electrolyte.^[^ ^156^ ^]^ In addition, their research indicated that the size and PL behavior of the as‐produced GQS could be altered by adjusting the current densities. Generally, the exfoliation strength will increase as the current density increases, resulting in a smaller size of GQSs.^[^ ^152^ ^]^

**Figure 6:**
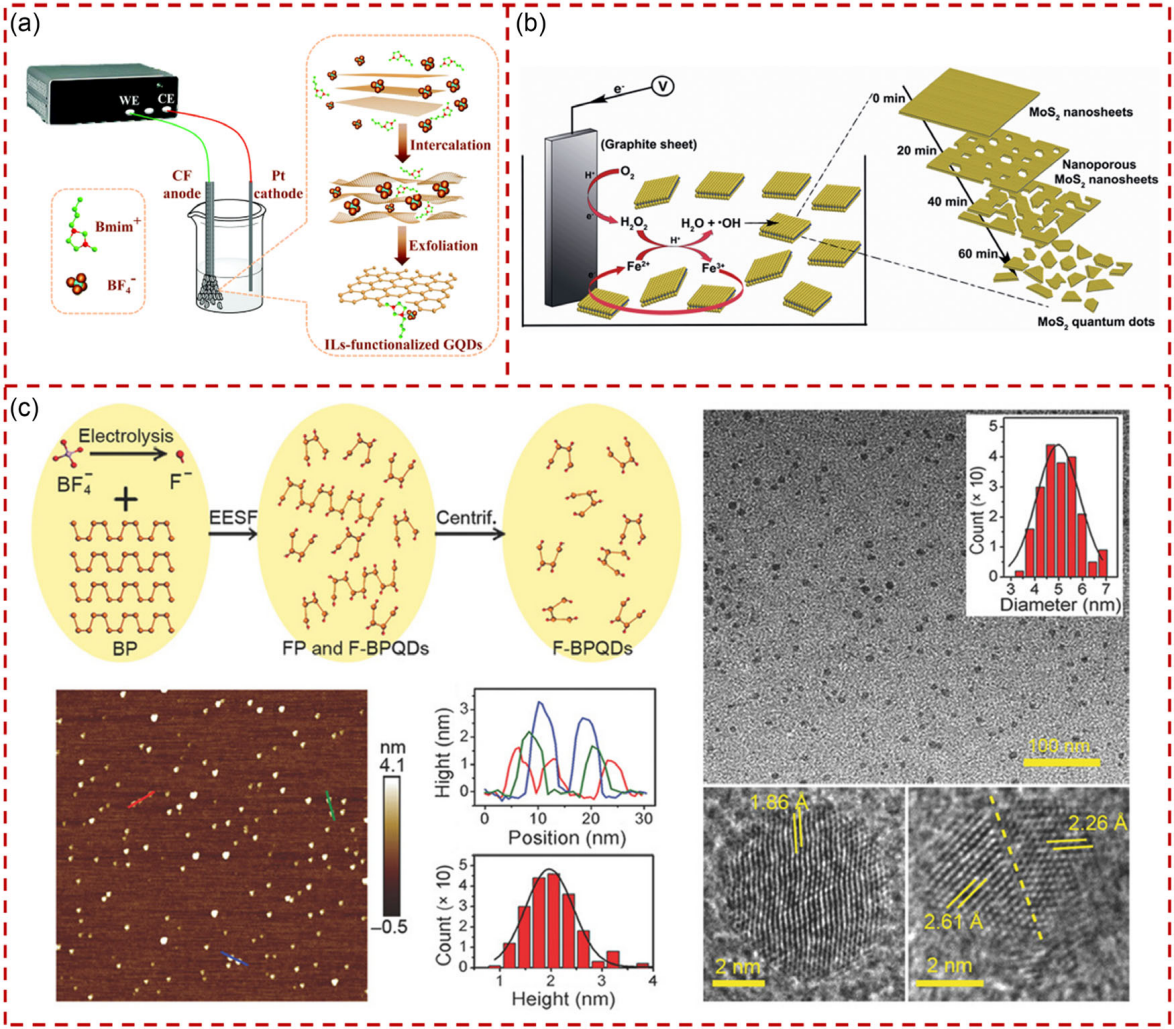
The production of quantum‐sized materials through electrochemical exfoliation. a) Schematic illustration of the electrochemical exfoliation process for the preparation of GQSs. Reproduced with permission.^[^ ^143^ ^]^ Copyright 2017, Royal Society of Chemistry. b) Schematic illustration of the exfoliation of bulk MoS_2_ for the production of nanoporous MoS_2_ NSs and MoS_2_ QSs by the electro‐Fenton process. Reproduced with permission.^[^ ^158^ ^]^ Copyright 2014, Royal Society of Chemistry. c) Schematic illustration of the synthesis of F‐BPQSs from electrochemical exfoliation and their corresponding AFM and TEM images. Reproduced under the terms of the CC‐BY Creative Commons Attribution 4.0 International license (https://creativecommons.org/licenses/by/4.0).^[^ ^159^ ^]^ Copyright 2018, The Authors, published by Wiley‐VCH.

The electrolyte was another important factor affecting the production of QSs during electrochemical exfoliation process.^[^ ^89^ ^]^ The ions in the electrolyte acting as electrochemical “scissors” were essential for the electrochemical exfoliation of QSs. Yang et al. prepared 3 nm GQSs with red PL emission via the electrolysis of graphite rod in an aqueous solution of 0.01 m K_2_S_2_O_8_.^[^ ^144^ ^]^ They found that significant red PL, very weak red PL, and no PL were observed when sodium persulfate, sodium sulfate, and potassium ferrite were selected as the electrolyte, respectively. The results indicated that the type of sulfate electrolyte has a crucial effect on the PL performances of the as‐produced GQSs. Shaijumon et al. reported that the luminescent MoS_2_ QSs with a narrow size distribution were controllably synthesized from bulk MoS_2_ in 1‐butyl‐3‐methylimidazolium chloride ([BMIM]Cl) or lithium bis‐trifluoromethylsulfonylimide (LiTFSI)‐based electrolyte.^[^ ^157^ ^]^ The lateral sizes of the MoS_2_ QSs produced using LiTFSI‐based electrolytes with concentrations of 0.1 and 1 wt% were 2.5 and 4.6 nm, respectively, while QSs produced using [BMIM]Cl‐based electrolytes with concentrations of 0.1 and 1 wt% were slightly larger, 2.8 and 5.8 nm, respectively. It was demonstrated that the size of the MoS_2_ QSs could be controlled by adjusting the composition of the electrolyte. The influence of water content in the electrolyte on the fabrication of QSs cannot be ignored during electrochemical exfoliation. Wang et al reported a large‐scale and controllable preparation of functionalized GQSs using carbon fibers (CFs) as anode and platinum wire as counter electrode in the 1‐butyl‐3‐methylimidazolium tetrafluoroborate.^[^ ^143^ ^]^ The schematic diagram of the electrochemical exfoliation of CFs is presented in **Figure** **6**a. The results showed that the ionic liquid with water content of 0, 15, and 30% generated blue‐, green‐, and yellow‐emitting GQSs, respectively, under irradiation at 365 nm. Such phenomenon was attributed to the different sizes of the obtained GQSs, with the average particle sizes of blue‐, green‐, and yellow‐emitting GQS being approximately 2, 3, and 4 nm, respectively. Meanwhile, a well‐designed electrochemiluminescence sensor was fabricated by using the obtained functionalized GQDs for the determination of pentachlorophenol with satisfactory sensitivity.

In recent years, the report on functional QSs prepared by electrochemical exfoliation assisted by other methods has been continuously increasing. Li et al. demonstrated that electrochemically induced Fenton (electro‐Fenton) reaction could be utilized to controllably generate hydroxyl radicals, which was essential for the fabrication of MoS_2_ QSs through exfoliating MoS_2_ nanosheets.^[^ ^158^ ^]^ The interaction between generated hydroxyl radicals and MoS_2_ nanosheets was investigated, revealing the gradual production of nanoporous MoS_2_ NSs and MoS_2_ QSs through etching of MoS_2_ nanosheets, as illustrated in Figure 6b. The as‐produced MoS_2_ QSs were with literal size of 5 nm and thickness of 0.7 nm. Such a structure showed enhanced PL and hydrogen evolution catalytic performances due to its sharply increased active site. Zhang et al. combined electrochemical exfoliation and synchronous fluorination methods to prepare highly selective fluorinated BPQSs (F‐BPQSs) with an average lateral size of 5.0 ± 2.0 nm and thickness of 2.0 ± 1.2 nm.^[^ ^159^ ^]^ The schematic diagram of the production of F‐BPQSs is shown in Figure 6c. The 1‐ethyl‐3‐methylimidazolium tetrafluoroborate was used as electrolyte for electrochemical exfoliation and fluorination reaction simultaneously. This study exhibited that the fluorination strategy imparted F‐BPQSs with excellent properties for effectively enhancing the environmental stability and eliminating electronic trap states of F‐BPQSs. Compared to previous BPQS preparation methods, this method could synthesize functionalized BPQSs in one step.^[^ ^160^, ^161^ ^]^ Electrochemical exfoliation was an effective method for the preparation of QSs or functionalized QSs, promoting the wide application of QSs in electronic devices, catalysis, sensors, and so on.^[^ ^143^, ^144^, ^155^, ^157^ ^]^ However, impurities during electrochemical exfoliation may be introduced into the material, which has adverse effects on the performance of the obtained QSs.^[^ ^162^ ^]^ In addition, the electrochemical exfoliation method is currently only used to prepare 2D QSs, which requires further development in the future.

### 3.5 Mechanical Exfoliation

**Figure 7:**
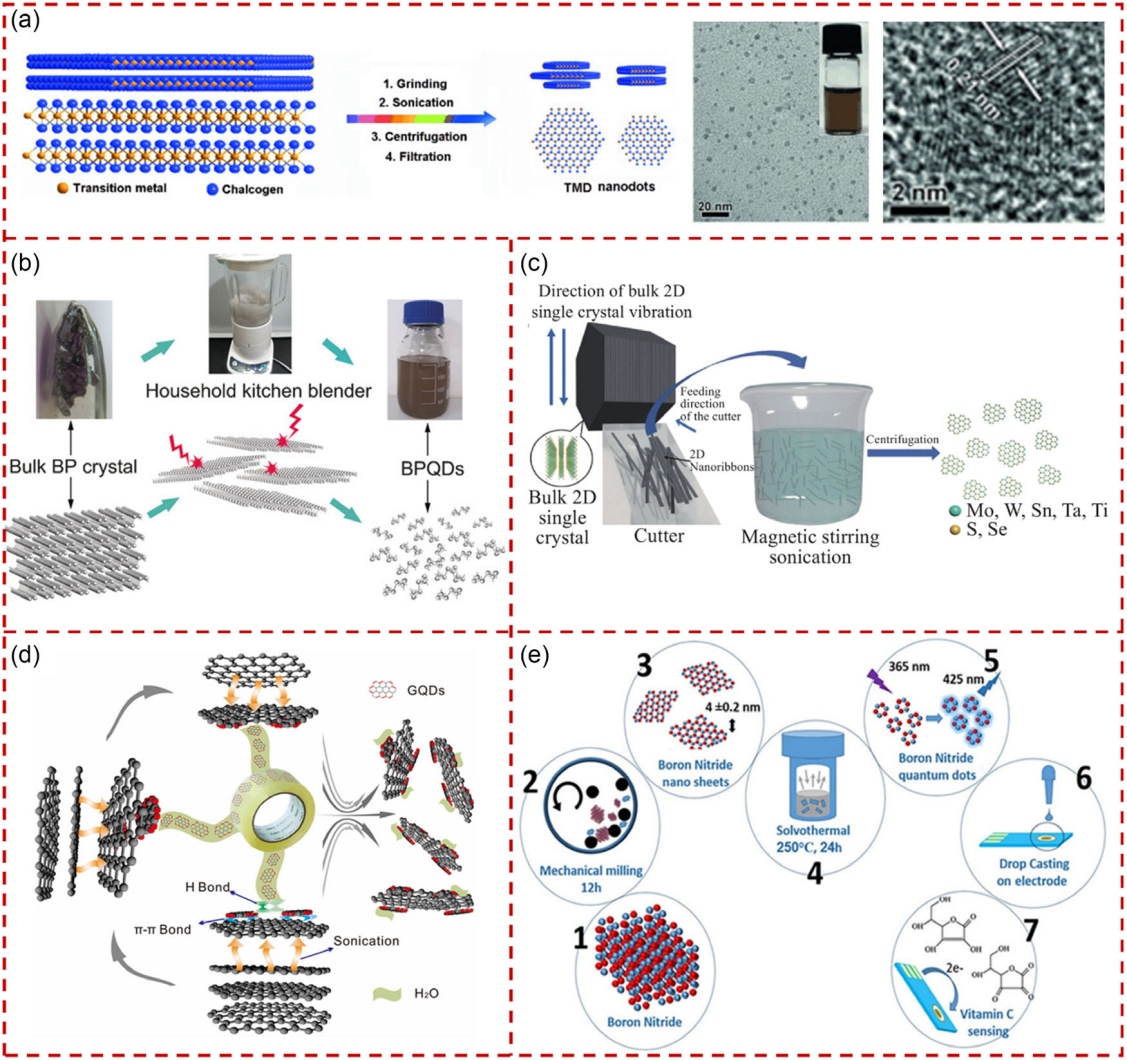
The production of quantum‐sized materials through mechanical exfoliation. a) The production of TMD QSs by grinding and sonication exfoliation. Reproduced with permission.^[^ ^164^ ^]^ Copyright 2015, Wiley‐VCH. b) Schematic diagram of producing BPQSs using a household kitchen blender. Reproduced with permission.^[^ ^165^ ^]^ Copyright 2016, Wiley‐VCH. c) Schematic diagram of producing TMD QSs by ultrathin section and liquid phase dissection. Reproduced with permission.^[^ ^166^ ^]^ Copyright 2020, Springer Nature. d) Schematic diagram of preparing GQSs by tape exfoliation. Reproduced with permission.^[^ ^167^ ^]^ Copyright 2019, Elsevier. e) Mechanochemical synthesis of exfoliated edge‐functionalized BNQSs. Reproduced with permission.^[^ ^170^ ^]^ Copyright 2018, American Chemical Society.

Mechanical exfoliation involves micromechanical cleaving, grinding, and ball‐milling, which could break the weak van der Waals forces between the layers of bulk layered materials by applying external force.^[^ ^147^, ^163^ ^]^ This method has been widely used for exfoliating various layered materials since Geim et al. pioneered mechanical exfoliation of graphene from graphite in 2004.^[^ ^4^ ^]^ Zhang et al. successfully produced a series of TMD QSs from their bulk materials by the combination of (wet) grinding and sonication exfoliation.^[^ ^164^ ^]^ The crystal structure of layered TMD and the process of preparing TMD QSs from bulk layered TMDs are shown in **Figure** **7**a. The bulk TMD materials were ground and sonicated twice in NMP at room temperature, then the TMD QSs were prepared followed by centrifugation and filtration with n‐hexane and chloroform. The TEM images showed that the size of as‐produced MoSe_2_ QSs was 2.7 ± 0.8 nm without aggregation, and the high‐resolution TEM (HRTEM) images indicated the high crystallinity of the QSs. However, the production yield of the TMD QSs was extremely low (below 1 wt%). There were many strategies for mechanical exfoliation that relied on various crushers. Sun et al. reported the production of BPQSs from bulk BP materials through utilizing a household kitchen blender.^[^ ^165^ ^]^ The highly turbulent shear forces generated by the blender caused layer‐by‐layer disintegration of large BP crystals, resulting in the exfoliation of a small amount of BPQSs. The disintegrating process of preparing BPQSs in dimethyl sulfoxide (DMSO) using a household kitchen blender is illustrated in Figure 7b. In this process, DMSO as a stable solvent with suitable surface energy could break the interaction forces between the BP interlayers. The average lateral size of the obtained BPQSs was 2.25 nm, with a thickness range of 0.58–1.45 nm, suggesting monolayered or double‐layered structure of BPQSs. Zheng et al. developed a general approach to fabricate high‐quality monolayer single crystal TMD QSs by directly ultrathin cutting and sonication treatment of bulk TMD single crystals.^[^ ^166^ ^]^ The fabrication process of TMD QSs is schematically shown in Figure 7c. The bulk crystals were cut by ultramicrotome at room temperature, then dispersed in the solvent for magnetic stirring and liquid exfoliation. A series of TMD QSs were obtained followed by centrifugation with the addition of hexane and chloroform. However, this method is limited by TMD single crystals, hindering its practical application.

The scotch tape stripping has been widely studied since it was first used to exfoliate graphite into graphene.^[^ ^4^ ^]^ Recently, Xu et al. proposed a similar scotch‐tape method to generate GQSs adsorbed GNSs, as shown in Figure 7d.^[^ ^167^ ^]^ This mechanical exfoliation process would not introduce structural defects to GNSs, guaranteeing their intrinsic physical properties (such as excellent electrical conductivity and long‐term stability). Frequently, mechanical exfoliation and other methods could be used together to obtain a wider variety of functionalized quantum‐sized materials.^[^ ^168^, ^169^ ^]^ For instance, Simchi et al. reported a green and efficient method based on mechanochemical exfoliation for fabricating hydroxylated BNQSs.^[^ ^170^ ^]^ The route for synthesizing BNNSs and BNQSs is shown in Figure 7e. Bulk h‐BN powders were first exfoliated into BNNSs through high‐energy ball‐milling for 16 h in ethanol. After ball‐milling and centrifuging, the obtained BNNSs were subjected to solvothermal treatment to prepare functionalized BNQSs. The as‐produced hydroxylated BNQSs were with an average lateral size of 4 nm and an average thickness of 2 nm. Although mechanical exfoliation has made much progress in preparing high‐quality monolayer 2D QSs, there remained shortcomings such as low yield and high cost.^[^ ^163^, ^167^ ^]^

### 3.6 Ball‐Milling

Ball‐milling, as an effective, environment‐friendly, economical, and reliable technology, has been widely used in experimental research and industrial production.^[^ ^171^ ^]^ Ball‐milling technology has significant advantages in preparing various downsized metals/alloys, oxides, carbides, and inorganic/organic compounds or mixtures.^[^ ^172^, ^173^ ^]^ In the field of nanotechnology, ball‐milling was applied for the fabrication of various nanomaterials such as carbon‐based nanomaterials, ceramic nanomaterials, and nanoalloy materials. To obtain extremely downsized nanomaterials, most efforts were committed to changing the parameters of ball‐milling, including increasing ball‐milling speed, extending ball‐milling time, and adjusting feed ratio. However, previous reports have shown that the size of nanomaterials prepared through ball‐milling was relatively large.^[^ ^174^, ^175^, ^176^ ^]^ Very few reports have produced quantum‐sized materials from layered materials through ball‐milling. Yang et al. prepared CQDs from activated carbon by high‐energy ball‐milling (HEBM). The mixture of activated carbon and alkaline reagent (KOH) was first pulverized by HEBM with stainless‐steel balls at 500 rpm for 50 h, then the CQD solutions were obtained after sonication, centrifugation, and filtration.^[^ ^169^ ^]^ In this process, the use of KOH will facilitate the exfoliation and etching of the bulk material, leading to nonintrinsic characteristics. Chen et al. exhibited a combined strategy to produce BPQSs through the combination of ball‐milling and shock process.^[^ ^177^ ^]^ First, the red phosphorus (RP) nanopowders were obtained through ball‐milling of bulk RP powder. After that, the as‐milled powders were induced phase transformation into BPQDs under transient high pressure and temperature. However, this method cannot be promoted owing to its low efficiency and high consumption.

**Figure 8:**
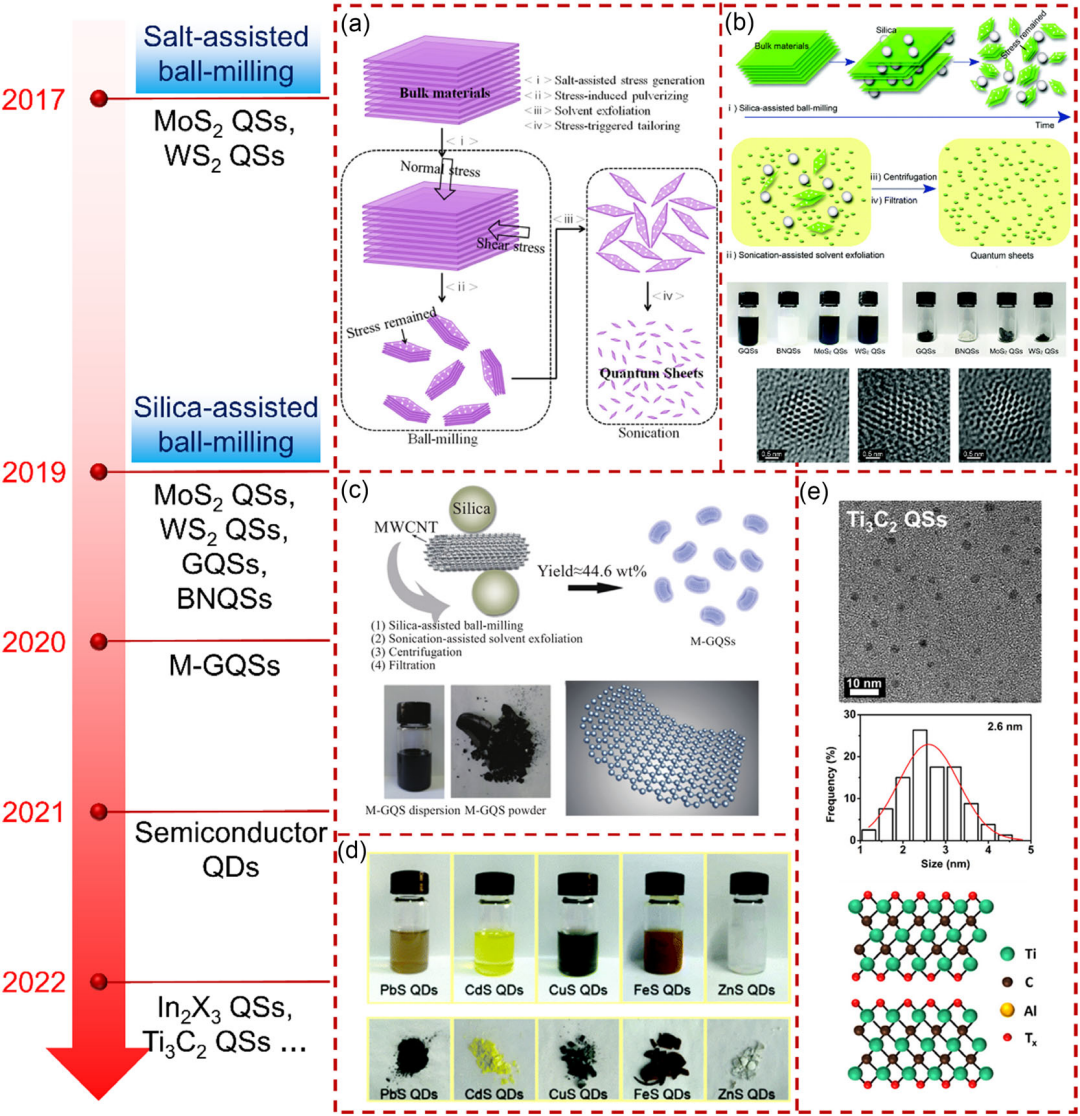
The production of quantum‐sized materials through assisted ball‐milling. a) Salt‐assisted ball‐milling and sonication‐assisted solvent exfoliation. Reproduced with permission.^[^ ^22^ ^]^ Copyright 2017, American Chemical Society. b) Silica‐assisted ball‐milling and sonication‐assisted solvent exfoliation. Reproduced with permission.^[^ ^23^ ^]^ Copyright 2019, Royal Society of Chemistry. c) Robust strategy for tailoring multiwalled carbon nanotubes into GQSs. Reproduced with permission.^[^ ^24^ ^]^ Copyright 2020, American Chemical Society. d) Photographs of the as‐produced QD dispersions and powders. Reproduced with permission.^[^ ^27^ ^]^ Copyright 2021, Royal Society of Chemistry. e) The TEM image and structure diagram of the as‐produced Ti_3_C_2_ MXene QSs. Reproduced with permission.^[^ ^29^ ^]^ Copyright 2022, American Chemical Society.

From the overview of top‐down production of quantum‐sized materials, most of the reported methods could only produce QSs from the layered materials with low breaking strength. Meanwhile, the current methods suffer from high cost and low yield. Evidently, the development of an innovative strategy for the universal and scalable production of quantum‐sized materials was inevitable and urgent. Fortunately, we have recently developed a general strategy toward the production of 2D QSs and 0D QDs from their bulk materials.^[^ ^22^, ^23^, ^24^, ^25^, ^26^, ^27^, ^28^, ^29^, ^30^, ^31^, ^178^, ^179^ ^]^ **Figure** **8** shows the achievements of our group in the production of quantum‐sized materials in recent years. The creative ball‐milling method not only revolutionized the ball‐milling technology with dual synergy effect by pushing the limit to the quantum scale but also exhibited the potential to establish a quantum‐sized material database/library based on identical protocols/criteria.

Our group has developed special (i.e., salt‐assisted ball‐milling) and general (i.e., silica‐assisted ball‐milling) methods to produce quantum‐sized materials.^[^ ^22^, ^23^ ^]^ During the salt‐assisted ball‐milling process, sodium chloride remained cubic crystallites with average sizes of approximately 1–3 μm.^[^ ^22^ ^]^ The calculated pressure on the 2D material surfaces was 4–36 GPa through the opposite faces of the sodium chloride crystallites. Therefore, intrinsic MoS_2_ and WS_2_ QSs with high production yield (20 wt%) were achieved through a sequential combination of salt‐assisted ball‐milling and sonication‐assisted solvent exfoliation of the bulk materials (with breaking strength of 16–30 GPa). The mechanism for the production of the QSs is shown in Figure 8a. The salt‐assisted ball‐milling induced normal and shear stress inside the bulk materials simultaneously. During this process, the chemical bond of bulk MoS_2_ and WS_2_, especially the covalent bond in the layer, will be extremely broken due to the torque and pressure. The subsequent sonication was powerful enough to break the remaining stress points so that intrinsic QSs were obtained. Unlike salt‐assisted ball‐milling, the pressure generated by silica‐assisted ball‐milling was extremely high (up to 1146 GPa) through circular contact (presumed radius of 100 nm). Such high pressure could effectively break the chemical bond of any known materials with the highest breaking strength of 130 GPa (i.e., monolayer graphene), thus producing quantum‐sized materials on a large scale. The schematic illustration of silica‐assisted ball‐milling and the obtained QS dispersions/powders with their corresponding HRTEM images are displayed in Figure 8b. A variety of quantum‐sized materials including GQSs, BNQSs, MoS_2_ QSs, and WS_2_ QSs with exceedingly high production yields (i.e., 35.5, 33.6, 30.2, and 28.2 wt%, respectively,) were produced from their bulk layered materials via the combination of silica‐assisted ball‐milling and sonication‐assisted solvent exfoliation. From the HRTEM, it should be noted that the as‐produced quantum‐sized materials possess intrinsic characteristics (perfect internal lattice, extremely exposed edges (no coating/functionalization, no ligands/surfactants, etc.)). Such a robust strategy enabled extremely high yield (44.6 wt%) production of M‐GQSs with intrinsic curvature and single‐crystalline characteristics, as presented in Figure 8c.^[^ ^24^ ^]^ In addition, a range of quantum‐sized materials, such as MoSe_2_ QSs, MoTe_2_ QSs, WSe_2_ QSs, WTe_2_ QSs, In_2_S_3_ QSs, In_2_Se_3_ QSs, In_2_Te_3_ QSs, Ti_3_C_2_ QSs, Ti_3_AlC_2_ QSs, SiQDs, PbS QDs, CdS QDs, CuS QDs, FeS QDs, and ZnS QDs (partially shown in Figure 8d,e), were successfully produced from their bulk materials (including layered and nonlayered materials).^[^ ^25^, ^26^, ^27^, ^29^, ^30^, ^31^ ^]^ The lateral size of the as‐produced quantum‐sized materials was basically between 2 nm and 5 nm. For 2D QSs, its thickness was between 0.5 and 1.6 nm, which was basically monolayer. For 0D QDs, its height was equivalent to their lateral size.

Our numerous studies demonstrated that silica‐assisted ball‐milling has great advantages in producing quantum‐sized materials.^[^ ^23^, ^24^, ^25^, ^26^, ^28^, ^29^, ^30^, ^31^, ^178^, ^179^ ^]^ On one hand, the method enables the intrinsic, universal, and scalable production of quantum‐sized materials. On the other hand, the method maximizes their intrinsic edge effects (nonequilibrium situation (e.g., broken lattices, unsaturated/dangling bonds, dynamic changes, etc.) and asymmetric environment). Such significant effects could be determinative to their extraordinary performances. Meanwhile, the intrinsic quantum‐sized materials have shown great significance in fields such as nonlinear optics,^[^ ^23^ ^]^ carrier dynamics,^[^ ^180^ ^]^ solar cells,^[^ ^181^ ^]^ and electrocatalysts.^[^ ^28^ ^]^ Our works have exhibited the great potential of the highly unified top‐down method (i.e., silica‐assisted ball‐milling and sonication‐assisted solvent exfoliation), which would undoubtedly boost the mass production and full exploration of quantum‐sized materials.

### 3.7 Chemical Treatment

**Figure 9:**
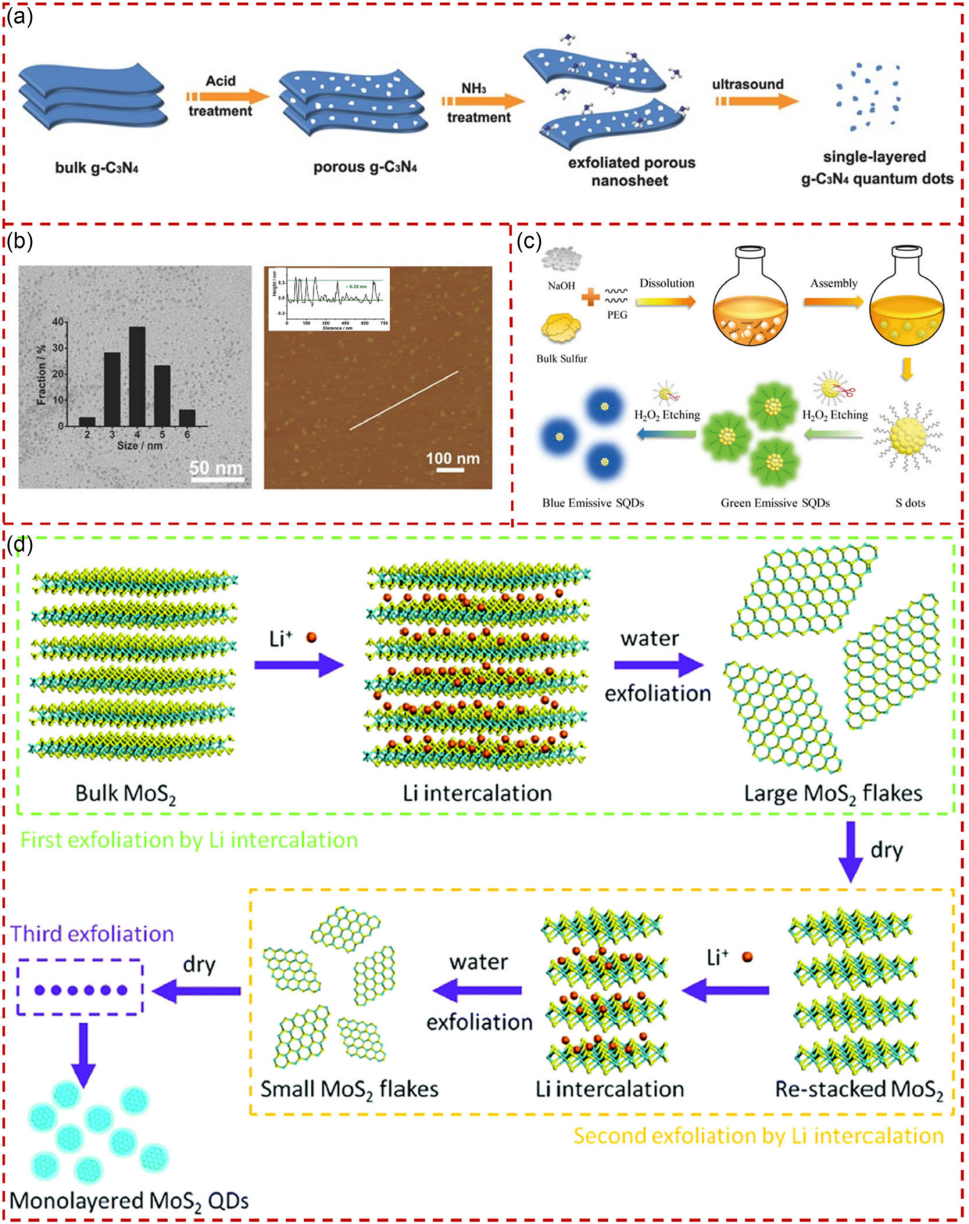
Production of quantum‐sized materials through chemical treatment. a) Schematic diagram of g‐C_3_N_4_ QSs by acid/alkali treatment and ultrasonic exfoliation. b) TEM and AFM images of the g‐C_3_N_4_ QSs. a,b) Reproduced with permission.^[^ ^186^ ^]^ Copyright 2014, Wiley‐VCH. c) Schematic diagram of the preparation for sulfur QDs via a H_2_O_2_‐assisted etching approach. Reproduced with permission.^[^ ^187^ ^]^ Copyright 2019, Wiley‐VCH. d) Schematic diagram of the fabrication procedure of MoS_2_ QSs employing multiexfoliation based on Li^+^ intercalation. Reproduced with permission.^[^ ^192^ ^]^ Copyright 2015, Elsevier.

The development of chemical treatment, such as the use of strong acids, strong alkalis, and strong oxidants to exfoliate bulk materials, provides another feasible way to obtain quantum‐sized materials.^[^ ^182^, ^183^, ^184^, ^185^ ^]^ Xie et al. reported an alternative approach for the fabrication of monolayer g‐C_3_N_4_ QSs via acid/alkali and sonication treatment, as displayed in **Figure** **9**a.^[^ ^186^ ^]^ First, porous g‐C_3_N_4_ was generated from bulk g‐C_3_N_4_ through acid treatment. Then, the as‐prepared g‐C_3_N_4_ was exfoliated into ultrathin NSs using hydrothermal treatment with NH_3_·H_2_O. Finally, the monolayer g‐C_3_N_4_ QSs were obtained from ultrathin porous g‐C_3_N_4_ NSs through sonication in water. The TEM and AFM images of the as‐fabricated g‐C_3_N_4_ QSs are shown in Figure 9b, in which the average lateral size of 4 nm and the thickness of 0.35 nm were derived for g‐C_3_N_4_ QSs. Rogach et al. reported a study in which sodium hydroxide (NaOH) and polyethylene glycol (PEG) were used to exfoliate bulk sulfur powder, followed by etching with H_2_O_2_ to prepare sulfur quantum dots (SQDs).^[^ ^187^ ^]^ The route of preparation of SQDs through an H_2_O_2_‐assisted top‐down etching is illustrated in Figure 9c. The bulk sulfur powders were first dissolved into small particles through the mixing PEG and bulk materials in an alkaline environment. Then, SQDs with strong green PL were prepared after the introduction of H_2_O_2_, and the green PL transformed into a very strong blue PL after successive injections of H_2_O_2_. This phenomenon was attributed to the fact that the H_2_O_2_‐assisted etching could control the size and surface etching degree of the obtained SQDs.

In addition, the research area of ion intercalation in bulk layered materials for producing QSs has grown rapidly.^[^ ^151^, ^188^, ^189^ ^]^ Metal ions (e.g., lithium ions (Li^+^), sodium ions (Na^+^)) typically possess small ion volumes and high reactivity, making them easy to insert into layered materials.^[^ ^190^, ^191^ ^]^ The ion intercalation weakens the in‐plane and out‐of‐plane bonding forces of the bulk material, which was beneficial for exfoliation to obtain downsized nanostructures. Du et al. reported a strategy for preparing monolayer MoS_2_ QSs by employing multiexfoliation based on Li^+^ intercalation of bulk MoS_2_.^[^ ^192^ ^]^ The schematic diagram of the fabrication process of MoS_2_ QSs is presented in Figure 9d. The third exfoliation based on Li^+^ intercalation made the MoS_2_ fragile and easy to break up, resulting in the formation of MoS_2_ QSs. The cutting mechanism may involve the complete breakup around the edges and defects during the reaction of Li_*x*_MoS_2_ with water and the following sonication process. Obviously, the biggest disadvantage of this method is that it is cumbersome and time‐consuming. Cyraic et al. introduced a cost‐effective platform for the production of MoS_2_ QSs by the ion intercalation without any external stimuli.^[^ ^193^ ^]^ The intercalation of Na^+^ ions into MoS_2_ layers and subsequent oxidative cutting reaction resulted in the formation of MoS_2_ QSs (with an average lateral size of 3.8 nm). However, the intrinsic properties of the material could be changed under the action of ions. For example, metal ion intercalation usually causes changes in the electronic structure and significant phase transitions of 2D materials.^[^ ^192^, ^194^ ^]^

The chemical exfoliation has been favored by most researchers due to its simplicity and ease of operation for the preparation of quantum‐sized materials.^[^ ^183^, ^195^ ^]^ However, the drawbacks of the chemical exfoliation were the waste liquid pollution in the preparation process. Moreover, the existence of certain structural defects in the obtained materials could lead to decline in their properties, thereby limiting its practical applications.^[^ ^89^, ^162^ ^]^

## 4 Conclusion and Prospect

A comprehensive overview of various top‐down strategies for the production of quantum‐sized materials was provided. We elaborated on several commonly used and well‐developed methods, including sonication exfoliation, laser ablation, heat treatment, electrochemical exfoliation, mechanical exfoliation, ball‐milling, and chemical treatment, meanwhile analyzed the advantages and disadvantages of each method. In particular, we emphasized the great significance of silica‐assisted ball‐milling in achieving universal and scalable production of intrinsic quantum‐sized materials.

Despite the encouraging progress that has been made in the top‐down strategy for producing quantum‐sized materials, there are still many challenges that need to be addressed. 1) It needs to build a complete quantum‐sized materials database on the same protocol/standard. However, this construction process is a long and arduous task that requires the participation of numerous researchers. 2) Accurate control over the size of materials is quite difficult to achieve via the top‐down method. It is necessary to realize the precise control of lateral size and thickness independently to explore the in‐plane and out‐of‐plane quantum confinement effects respectively. 3) The existing production methods are difficult to achieve atomic control of quantum‐sized material geometry/edge structure. The materials with atomic control of the surface/edge structures could be used to explore the surface/edge effects, as well as their interactions. 4) There is limited research on the production or design of quantum‐sized materials with specific properties/functions through top‐down methods, including heterostructured, hybrid, alloyed, and composite materials. Therefore, we should accelerate the production of functional materials with superior performance to achieve significant breakthroughs in industrial applications. 5) Due to the probably equal number of internal and surface/edge lattices in quantum‐sized materials, there is a great potential for quantum‐sized materials in phase engineering, interface engineering, defects engineering, doping, modification, and electronic state regulation. 6) The safety and benefits during the production process need to be considered. For example, the fabrication processes may be expensive and time‐consuming, which cannot guarantee the production on a large scale. The preparation processes may use or produce toxic and harmful substances, which poses risks to researchers and the environment. Therefore, it is crucial to develop effective, safe, reliable, and sustainable methods for producing quantum‐sized materials.

With the progress of science and technology and the efforts of researchers, we are optimistic about the application prospects of quantum‐sized materials and ready to face the great challenges currently present in the fields. Meanwhile, quantum‐sized materials are expected to play an irreplaceable role in metamaterials, optoelectronic devices, detectors and sensors, environment treatment, energy and catalysis, biological imaging, and other fields.

## Conflict of Interest

The authors declare no conflict of interest.
